# Developing the professional knowledge of librarians through a webinar series

**DOI:** 10.5195/jmla.2025.2071

**Published:** 2025-10-23

**Authors:** Katie Pierce Farrier, Sandra Desjardins, Laura Haygood

**Affiliations:** 1 katie.pierce@uta.edu, University of North Texas Health Science Center, Fort Worth, TX; 2 sandra.desjardins@library.tmc.edu, Texas Medical Center Library, Research & Instruction Librarian, Houston, TX; 3 laura_haygood@brown.edu, Brown University Library, Health Sciences Librarian for Public Health, Providence, RI

**Keywords:** Career development, continuing education, early career librarian, ECLI, emerging librarian, health science librarian, information overload, library professional, medical librarian, onboarding overload, professional development, sense of community, transitioning librarian

## Abstract

**Background:**

The Early Career Librarians Initiative of the South Central Chapter of the Medical Library Association (ECLI) offered a webinar series that addressed topics of interest to new professionals such as networking, goal setting, and salary negotiation. Additionally, the ECLI assessed participant feedback on the series through a program evaluation survey.

**Case Presentation:**

ECLI partnered with the Network of the National Library of Medicine (NNLM), Region 3, to offer six webinars over the course of two years. Attendees were asked to complete a survey. Quantitative results were analyzed, and qualitative free-text responses were thematically coded. A total of 567 people attended the webinars, and 154 completed the survey. Four major themes emerged as the most useful aspects of the webinar series: practical tips, encouragement, and real-life experience.

**Conclusion:**

Early career librarians often feel overwhelmed and are interested in guidance on career planning and building professional soft skills. This highly attended webinar series and positive evaluation feedback, demonstrates the value of providing accessible online professional development opportunities for early career and transitioning librarians, offering valuable information and support in key areas of need.

## ACRONYMS

Continuing Education **(CE)**; Curricula Vitae **(CVs)**; Early Career Librarians Initiative of the South Central Chapter of the Medical Library Association **(ECLI)**; Library and Information Science **(LIS)**; Network of the National Library of Medicine **(NNLM)**; Research Electronic Data Capture **(REDCap)**; South Central Chapter of the Medical Library Association **(SCC MLA)**.

## BACKGROUND

The onboarding process presents a significant investment of resources for organizations seeking to effectively integrate new members. In their article on identifying best employee onboarding practices in ACRL libraries, Graybill et al. [1] found that onboarding programs could vary between one week to six months. Novice health science librarians have more expansive onboarding needs. In addition to typical organizational training and office logistics, these librarians may need support with navigating health information resources, tenure and promotion procedures, and understanding the complex world of academia. Within this time frame, employers strive to equip new hires with a comprehensive knowledge base and essential job-related skills. This can necessitate the introduction of a substantial volume of information and the completion of tasks with established deadlines. Consequently, a recent study by Allen et al. [2] about utilizing lived experiences to improve onboarding in an academic library documented a phenomenon of “information overload,” where new employees felt overwhelmed due to the multifaceted demands placed upon them during this introductory period.

Despite an organization's efforts to optimize the volume and nature of disseminated information, often, a gap remains. This situation is especially true for early-career professionals, who not only lack institutional knowledge but are also unsure of ways to organize obtained information or how to discover resources that can help them fill those gaps. This challenge is particularly acute in specialized fields like health sciences librarianship.

Frequently, library school is not enough to adequately prepare new graduates to take on the nuanced professional roles within health sciences librarianship. “I didn't learn that in library school” is an unfortunate common refrain among new or early career librarians. In a 2023 survey conducted by the Association of Academic Health Sciences Libraries (AAHSL), 17.8% of library staff had less than five years of experience [3]. Additionally, there are so many paths and variables in librarianship that it's impossible to learn it all, and health science information tracks aren't available in every Library and Information Science (LIS) program [4]. More experienced professionals with diverse backgrounds, such as public, school, or academic librarianship, also notice a steep learning curve during their initial forays into biomedical sciences.

Myers and Rodriguez [5] surveyed health science librarians with less than five years of experience and asked them about their self-perceived career preparedness and professional competencies, and how they acquired that knowledge. The results of that survey indicated that early career professionals rely heavily on Medical Library Association (MLA) and Continuing Education (CE) opportunities to obtain professional competencies [5]. MLA offers a plethora of training opportunities and specialization tracks, such as Consumer Health Information, Systematic Reviews, and Data Services [6], but not every librarian has access to the funding needed to participate. Regardless, early career librarians still lack sufficient guidance to navigate the many training and education opportunities available.

While academic universities may have hundreds of employees, health sciences libraries typically have smaller staff, often only employing an average of 12 full-time librarians [3]. When looking for a peer with similar professional interests, goals, and career trajectories, it may be hard for early professionals to find role models or colleagues encountering similar obstacles. Connecting with other librarians across institutions, or even states, can help build people's professional networks and gives them insight into experiences they might not have access to otherwise.

Recognizing the need for a general space where resources could be shared and where early career professionals can engage and grow, Laura Wright and Laura N. Haygood co-founded the Early Career Librarians Initiative (ECLI) [7], a group geared toward providing information and opportunities for health sciences library students and early-career librarians. This group was initially formed within the South Central Chapter of the Medical Library Association (SCC MLA) but quickly grew to include groups in other chapters. Beyond its initial focus on providing information and opportunities, ECLI fosters professional networking and creates a shared space for librarians to build community, explore, and grow their careers.

While traditional learning opportunities like library school and continuing education programs address essential competencies, they often do not adequately prepare librarians for the practical realities, or “soft skills” necessary to navigate their careers. Cultivating a network of peers, as advocated by Bartley, Simuel, and Williams [8], can be crucial for professional success, as many of these interactions offer a space for colleagues to offer guidance and best practices for soft skills, like managing one's time, organizing information, and understanding the important, unspoken nuances of workplace wellness [9]. ECLI recognizes these needs by fostering a community where early career professionals can connect with colleagues who share similar interests and aspirations. This sense of community is further enhanced through their webinar series, which offers guidance and instruction from experienced professionals on topics specifically requested by ECLI members.

Formal and informal mentoring, continuing education, and self-teaching are all ways that health science librarians build competencies and skills [4]. The ECLI webinar series seeks to combine these facets of professional development by gathering feedback from ECLI members about topics of interest, offering guidance and instruction from experienced professionals, and then sharing the information widely. This article analyzes survey feedback collected from the webinar series.

## CASE PRESENTATION

ECLI aims to create more opportunities for new and early-career librarians and to grow as health information professionals. To address the identified shortage of professional development resources tailored to LIS students and early career health sciences librarians, the ECLI undertook a collaborative initiative to develop a webinar series. A rigorous planning process ensued, with biweekly meetings held to determine the schedule, roles, and responsibilities for the webinar series. Key considerations included topic selection aligned with identified knowledge gaps, speaker recruitment strategies, and developing a robust evaluation framework.

ECLI formed a collaborative relationship with the Network of the National Library of Medicine (NNLM). The partnership aligned with NNLM''s goals of expanding outreach and engaging the next generation of health science librarians [10]. NNLM Region 3 provided the platform to host the webinars, the infrastructure to create a registration page and track registration and attendance, and standardized surveys to solicit feedback from attendees. ECLI was responsible for finding and recruiting speakers, coordinating and marketing the events, and facilitating the webinars.

Participants were encouraged to complete a short evaluation survey at the end of each webinar (see Appendix B). The survey was a part of a quality assurance/improvement and program evaluation project from the NNLM. The Extramural Program of the National Library of Medicine approved it for this use and deemed it exempt from IRB approval. NNLM National Evaluation Center located at the Galter Health Sciences Library and Learning Center, Northwestern University, Feinberg School of Medicine in Chicago created the survey using Research Electronic Data Capture (REDCap) [11]. The survey questions were based on a 5-point Likert scale, and respondents were asked if they agree or disagree with each statement. Aggregated data were exported from Redcap into an Excel file to be analyzed.

An inductive thematic analysis (Braun and Clarke) [12] was done on the free-text responses. Responses to the question “What was the most useful aspect of this training and why?” and “Please tell us more about how you plan to use the information gained in this training” were reviewed and coded. Codes were grouped into four main themes: practical tips, resources, encouragement, and real-life experience were reviewed and refined through an iterative process.

## RESULTS

ECLI has offered a series of six webinars, featuring twelve guest speakers. All webinars were free and open to anyone interested in attending. Each webinar lasted an hour and a half, with speakers encouraged to reserve the last thirty minutes for questions, creating ample time and space for attendees to ask in-depth questions and learn from the perspective of more experienced, impartial professionals. At the end of each session, attendees were asked to complete a feedback survey. A total of 981 people registered for the webinars, with 567 attending at least one session. Among these, there were 446 unique attendees. In the first year of the webinar series, 240 people registered, and 212 attended; in the second year, 741 registered, and 355 attended. All webinars were recorded and shared on NNLM's YouTube page. As of January 27, 2025, the webinar recordings have been viewed 2,149 times. Please see Appendix A for full webinar details and attendance.

The webinar 2.1 “Integrating Research, Publishing, and Presenting Into Your Career,” had the highest engagement, with 143 attendees. The webinar 1.2 “Goal Setting (And Success in Achieving Them!) had the lowest engagement, with only 48 attendees.

The ECLI and NNLM Region 3 are both regional chapters of larger organizations with overlapping coverage areas. Together, they cover eight states (AR, LA, KS, MO, NE, NM, OK, and TX). The first year of the webinar series reached 35 states, including D.C., with the most attendees from Texas (24), Louisiana (13), and Arkansas (10); the second year reached 43 states, plus Puerto Rico and Washington, D.C., with the most attendees from Texas (51), California (29), and New York (13).

**Figure 1:**
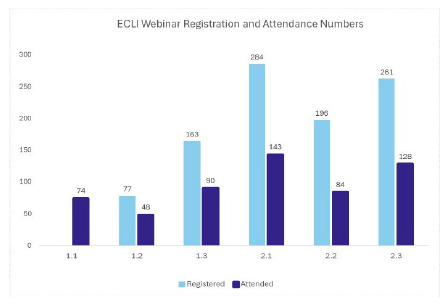
Attendance and registration for both years of the webinar series.

**Figure 2:**
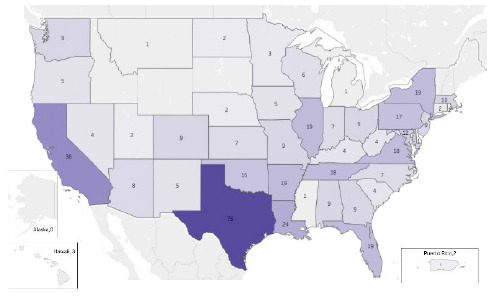
Geographic map of attendance.

**Figure 3:**
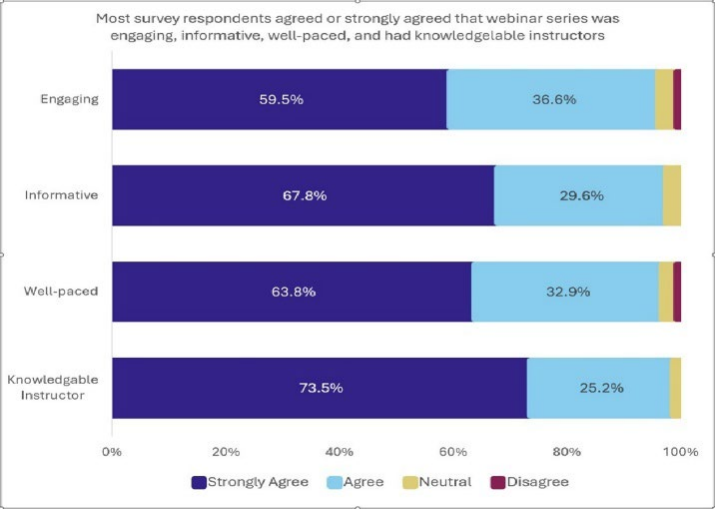
Survey Responses.

Note: There were no “strongly disagree” responses.

An evaluation survey link was shared by the session hosts at the end of each webinar. The overall survey response rate was 28.73% with 154 completed surveys, with 64 completed in the first year and 90 completed in the second. We combined data from all surveys for analysis.

No demographic information was collected from attendees, but the series was shared through targeted marketing to LIS schools and faculty, medical library directors, and groups aimed at serving early career librarians.

Most survey respondents agreed or strongly agreed that the webinars were engaging, informative, well-paced, and led by a knowledgeable instructor.

### Themes

Overall, four themes were identified as most substantive from the open-ended questions “What was the most useful aspect of this training and why?” and “Please tell us more about how you plan to use the information gained in this training.” These themes align with the mission of ECLI to engage new library professionals and help them grow professionally.

### Practical Tips

Practical tips focused on quick, actionable advice or guidance that attendees could readily implement in their own professional lives. Such tips often included basic information that more seasoned professionals take for common knowledge, like how long a cover letter should be, layout of a research poster, and how to get involved with professional organizations.

*“The session provided great suggestions and advice about how to engage him/herself and connect with others in MLA.”*

In the library profession, collaborations, mentorships, and colleagues often span multiple institutions. People benefit from guidance on how to seek out and foster those relationships as well as more practical tips for dealing with day-to-day job responsibilities. Connecting with coworkers and colleagues at other institutions can help combat burnout and feelings of being overwhelmed [13].

### Resources

Attendees benefited from sharing resources with each other through the chat and the speaker presentations. People said that the shared links, recommended books, speaker slides, job postings and salary databases were useful.

### Encouragement

*“The most useful aspect of this webinar was hearing about all of the opportunities we have to grow in our careers as health science librarians and knowing that we are supported.”*

Starting a new career can be daunting. Librarians often face imposter syndrome, especially in the first few years of their career [13, 14]. Imposter syndrome can affect confidence and willingness to try new things [14, 15, 16]. Reaching out to people and building a supportive network are ways to combat imposter syndrome [15].

*“I am attempting to start a new program within my library and this presentation helped me stay encouraged for the task at hand”*

Sometimes all people really need is a little encouragement. Library leadership can support new librarians to participate in groups such as ECLI as a way to expand professional networks and mature professionally.

### Real-Life Experience

Respondents appreciated the opportunity to see “real life” examples of other people's Curricula Vitae (CVs), their work processes, and methods for publishing and presenting. This behind-the-scenes look doesn't take the place of mentorship or formal guidance, but it shows early career professionals' different ways to approach problems and opportunities. Hearing other people's success stories and how they built their careers can light the way for others to follow in their footsteps.

*“LIS research can be so unique in the way we conduct, perform, and present as compared to “hard” sciences that I really appreciated hearing straight from peers.”*

Libraries often have smaller staff sizes [3]. When looking for a peer with similar professional interests, goals, and career trajectories, it may be hard for early professionals to find role models or comrades encountering similar obstacles.

## DISCUSSION

The webinar series was well-attended and quickly attracted an audience. While the webinar series was offered by SCC's ECLI and NNLM Region 3, the need for interprofessional support, advice, and guidance is not limited to early career professionals in their regional states. People across the nation attended the webinars, and ECLI continues to form and grow across other MLA chapter organizations.

Attendance dipped in April, possibly because of co-occurring academic and conference events such as MLA, ACRL, TLA, and final exams. When asked informally during regular ECLI meetings, members agreed that April was a busy month and made it challenging to attend webinars.

The ECLI selected topics relevant to its members, focusing on career beginnings and early success. These topics differed from other professional development opportunities by focusing on soft skills, hiring and promotion processes, and professional reputation building rather than hard skills like literature searching, evidence synthesis, collection development, or other library-specific skills. At 90 minutes long, the sessions were longer than a traditional webinar to allow more time for discussion and questions. This format allowed participants to ask more anecdotal questions and solicit advice. One participant summed it up nicely when they said, “It was nice to almost be in conversation with the two speakers as they shared information but also personal examples and advice.”

These more informal conversations were a crucial part of the webinars and provided a learning opportunity that might not otherwise be available to early career librarians.

## CONCLUSION

Onboarding is a resource-intensive process, especially when it involves introducing large amounts of information to employees new to specialized fields. New and transitioning health sciences librarians, for example, are particularly susceptible to “onboarding overload.” This phenomenon encompasses learning how to handle all the relevant information pertaining to their job. Nuanced soft skills that allow them to function more effectively and fulfill all the requirements of becoming an essential liaison to their respective school or hospital. Unfortunately, most library school programs do not fully equip graduates with the tools and knowledge necessary to be successful in all these endeavors, which may lead to librarians feeling overwhelmed, stressed, anxious, and underprepared.

Moreover, experienced librarians may not always recall the challenges they faced early in their careers, especially as information and resources continue to evolve. Novices learn differently than experts and approach problems differently. [17]. Hence, it's crucial to encourage new and transitioning librarians to build networks of peers with shared interests and similar experience levels through groups like the ECLI, which offer a space for resource sharing and professional development.

Mentorship, continuing education, and self-teaching are all valuable resources that can help enhance soft skills and deepen understanding of core competencies. This webinar series aimed to combine these elements by offering guidance and instruction from experienced professionals, and it successfully reached a large audience and interest in both the series and initiatives like the ECLI. Results from the survey support the conclusion that the webinar series is helpful and addresses a topic of need and interest. To meet the strong demand for career development and professional skills among new LIS professionals, ECLI will continue its commitment to providing this valuable service for years to come.

The NNLM National Evaluation Center created the feedback survey and its use was required as part of the partnership with NNLM. The survey could not be altered, but it had some inherent limitations. The survey did not capture demographic data such as years of experience, library type, or job titles. This limited our ability to verify if the people attending the webinar series reflected the intended audience.

The survey was designed for educational offerings aimed at evaluating and using biomedical resources. As a result, several survey questions were unrelated to the webinar series content. Over half the respondents put “not applicable” or left the question blank for questions related to an increase in skills for finding, using, and evaluating health information resources and datasets.

Participants self-selected to attend the webinar series, which could lead to a bias of more positive results. Respondents reported that the webinar helped advance their careers, but we lack a reliable way of measuring that outcome. Evaluating the long-term impact of informal training was beyond the current scope of the project.

## IMPLICATIONS FOR FUTURE PRACTICE & POLICY

Library managers and directors should encourage early career librarians to participate in groups, such as ECLI, which help new professionals establish wide support networks. Mentors and leaders should be aware that in addition to training focused on technical skills, early career professionals can also benefit from guidance on soft skills, career planning, and hiring practices.

## Data Availability

Data associated with this article cannot be made publicly available because they contain personally identifiable information. Access to the data can be requested from the NNLM National Evaluation Center located at Galter Health Sciences Library and Learning Center, Northwestern University, Feinberg School of Medicine in Chicago which facilitates the survey and corresponding data. https://www.nnlm.gov/about/centers/nec. Email nec@northwestern.edu.
