# Supplementary material for: Developing the professional knowledge of librarians through a webinar series
Source: J Med Libr Assoc. 2025 Oct 23;113(4):336–41. doi: 10.5195/jmla.2025.2071 (PMC12604059; doi:10.5195/jmla.2025.2071)
Supplement: Supplementary file 1 — Appendix A [file jmla-113-4-336-s01.docx]

Appendix 1: Blinded Webinar Details and themes

TABLE 1: Registration, Attendance, and YouTube views of each webinar. Note: Registration for Session 1.1 was not captured.

| **Session** | **Date** | **Title** | **Speakers** | **Objectives** | **Registered** | **Attended** | **YouTube**  **Views** |
| --- | --- | --- | --- | --- | --- | --- | --- |
| 1.1 | Jan 2023 | Want to Be a Medical Librarian? How to Land Your First Job! | [Blinded] | - Understand the components of an academic interview (presentation and other meetings) - Identify the elements of an academic CV and structure content accordingly, differentiate between a CV and a resume - Writing cover letters for the job description - Relate transferable skills - Distinguish the differences between hospital and academic hiring   ·     Preparing for phone/virtual interview | Registration for this event was not captured | 74 | 1200 |
| 1.2 | April 2023 | Goal Setting (And Success in Achieving Them!) | [Blinded] | - Setting goals and working toward meeting them - Building a professional network to support your goals (finding collaborators) - Developing skills to meet your goals (places to seek professional development) - Applying for grants and other funding (NNLM grants, meeting travel awards, etc.). | 77 | 48 | 163 |
| 1.3 | July 2023 | Creating a Road Map for Career Success: Applying for Tenure, Promotion, or AHIP | [Blinded] | ·     Define relevant terminology  ·     Differentiate and compare the tenure, promotion, and AHIP processes, benefits & drawbacks  ·     Summarize the timelines involved and plan for application processes  ·     Organize necessary documentation  ·     Identify appropriate mentors  ·     Utilize available AHIP funding support | 163 | 90 | 234 |
| 2.1 | Feb 2024 | Integrating Research, Publishing, and Presenting Into Your Career | [Blinded] | - Identify strategies for beginning the research process   ·     Understand important considerations in the current publishing and academic environments and ways to navigate them  ·     Learn steps and skills to take your work from submission to presentation | 284 | 143 | 230 |
| 2.2 | April 2024 | From Wallflowers to Power Players: Mastering the Art of Networking | [Blinded] | - Building your professional network - Peer to peer networking - Building community partners - Networking at conferences | 196 | 84 | 186 |
| 2.3 | July 2024 | Know Your Worth: Mastering the Art of Salary Negotiation | [Blinded] | - Articulate the Value of Effective Salary Negotiation - Research and Analyze Librarian Salary Benchmarks - Identify and Quantify Personal Skills and Achievements - Develop a Negotiation Strategy and Communication Techniques - Evaluate and Negotiate a Competitive Compensation Package - Examine Student-focused Negotiation Tips | 261 | 128 | 136 |

TABLE 2: Themes, occurrences, definitions, and examples.

| **Theme** | **Definition** | **Attendee Reflection** | **Frequency** |
| --- | --- | --- | --- |
| **Practical Tips** | Concrete, actionable advice or guidance that attendees can readily implement in their own professional lives. This includes step-by-step instructions, how-to guides, and clear takeaways that translate into real-world application. | “It broke down the different steps of research and publishing, what is and isn't research, and how to go about writing a manuscript. It's such a daunting process that I feel like it's difficult to know where to start, and this webinar at least told me where to start. I also liked the tip about not including abstracts in poster presentations.” | 30 |
| **Resources** | Supplementary materials provided to enhance learning and support further exploration of the topic. This includes presentation slides, handouts, curated lists of links, and relevant bibliographic references. | “Information on external websites to further research.” | 27 |
| **Encouragement** | Expressions of support, motivation, and inspiration that instill confidence and empower attendees to pursue their professional goals. This includes messages that foster a sense of capability, resilience, and a belief in one's potential. | “There was a lot of useful information here! If I had to pick just one item, I would say the empowerment that comes along with understanding a salary negotiation as a conversation rather than a competition. Once you are able to let go of this competitive aspect, you understand that there is no downside to advocating for yourself in a professional and respectful manner.” | 24 |
| **Real-life Experience** | Instances where attendees specifically mentioned the value of learning from the presenters' personal experiences, anecdotes, or real-life examples. This includes references to insights gained from peers or colleagues sharing their professional journeys. | “I liked how the presenters’ showed aspects of their career that focus from the beginning to the end, [it] felt very relatable and possible to integrate into my own work.” | 21 |
| **Career Trajectory** | Statements indicating that attendees intend to use the information gained to make informed decisions about their career paths, explore new opportunities, or take concrete steps to advance in their careers. This includes mentions of seeking mentorship, pursuing grants, or developing specific skills. | “I'll be re-evaluating my professional track and making sure I am on target for my career goals” | 25 |
| **Sharing** | Expressions of intent to share the information or resources from webinars with others, including colleagues, students, or co-workers. This demonstrates a desire to disseminate knowledge and contribute to the professional growth of their peers. | “I hope to share some knowledge learned here with my peers so that they may benefit from it as well.” | 9 |
| **Complimentary** | General expressions of praise or positive feedback about the webinar without providing specific details or insights. This includes vague compliments or overall endorsements of the session. | “The info was so helpful!” | 7 |
| **Ambiguous** | Responses that merely reiterate the webinar content, title, or description without offering any further explanation, elaboration, or personal reflection. These responses lack specific details or insights into how the information was perceived or applied. | “Elevator speech.” | 4 |
