# Supplementary material for: Developing the professional knowledge of librarians through a webinar series
Source: J Med Libr Assoc. 2025 Oct 23;113(4):336–41. doi: 10.5195/jmla.2025.2071 (PMC12604059; doi:10.5195/jmla.2025.2071)
Supplement: Supplementary file 2 — Appendix B [file jmla-113-4-336-s02.docx]

# Survey Questions

[Blinded]

## Survey

1. Please indicate your level of agreement with the following statements about the [blinded]
   training that you attended. The [blinded] training that I attended (was)... Rated on a five-point Likert scale (Strongly disagree, Disagree, Neutral, Agree, Strongly agree).
   1. Engaging
   2. Informative
   3. Well-paced
   4. Led by a knowledgeable instructor
   5. Used examples and/or images reflecting diverse populations
2. Since you disagreed or strongly disagreed that this training was well-paced, please indicate if you found the pacing to be too fast or too slow.
   1. Too fast
   2. Too slow
3. If you disagreed or strongly disagreed with any of the items above, please share your feedback on opportunities to improve and/or address concerns regarding the training.
4. To what extent did this training increase your knowledge in the following areas? Rated on a four-point scale (Not at all, Somewhat, To a great extent, Not applicable).
   1. Learning about new resource(s) to explore and use
   2. Increasing knowledge of familiar resources
   3. Increasing understanding/awareness of specific audiences or populations
5. What was the most useful aspect of this training and why?
6. What suggestions do you have to improve this training (e.g., more/less discussion of particular topic areas, feedback on accessibility, usefulness, ease of understanding, etc.)
7. On a scale of 1 to 10, how likely are you to: 1 (Not at all likely) to 10 (Extremely likely)
   1. Attend other trainings offered by [blinded]
   2. Recommend [blinded] trainings to one or more colleagues.
8. What actions do you plan to take as a result of the training? Rated on a three-point scale (Yes, No, Not applicable).
   1. start using new resources or tools
   2. use more features of familiar resources or tools
   3. create or update an information resource
   4. share skills or resources learned with others
   5. apply for funding
   6. explore more advanced related topics
   7. plan a program, class, or activity
   8. other (please specify below):
9. Please tell us more about how you plan to use the information gained in this training.
10. How will this training impact your workplace and career? I expect to: Rated on a three-point scale (Yes, No, Not applicable).
    1. work more efficiently
    2. complete new tasks
    3. support my end users'/patrons' needs more effectively
    4. engage new audiences or populations
    5. advance my career
    6. other (please specify below):
11. What topics would you like to learn about in future trainings?
12. Do you want to receive [blinded] credit for this training? (Yes or No).
